# Multisequence Quantitative Magnetic Resonance Neurography of Brachial and Lumbosacral Plexus in Chronic Inflammatory Demyelinating Polyneuropathy

**DOI:** 10.3389/fnins.2021.649071

**Published:** 2021-07-23

**Authors:** Xiaoyun Su, Xiangquan Kong, Osamah Alwalid, Jing Wang, Huiting Zhang, Zuneng Lu, Chuansheng Zheng

**Affiliations:** ^1^Department of Radiology, Union Hospital, Tongji Medical College, Huazhong University of Science and Technology, Wuhan, China; ^2^Siemens Healthineers, Shanghai, China; ^3^Department of Neurology, Renmin Hospital of Wuhan University, Wuhan, China

**Keywords:** magnetic resonance neurography, diffusion tensor imaging, Polyradiculoneuropathy, chronic inflammatory demyelinating, plexus, contrast

## Abstract

**Background and Purpose:**

Chronic inflammatory demyelinating polyneuropathy (CIDP) is an uncommon demyelinating disorder. Although treatable, it is difficult to diagnose. The purpose of this study was to evaluate the diagnostic performance and abnormalities of plexus via quantitative multisequence magnetic resonance neurography (MRN) for CIDP.

**Methods:**

Brachial and lumbosacral (LS) plexus of 37 CIDP patients and 37 age- and gender-matched controls were examined by using multisequence MRN. Nerve diameter, nerve-to-muscle T2 signal intensity ratio (nT2), contrast-enhanced ratio (CR), fractional anisotropy (FA), and apparent diffusion coefficient (ADC) were determined in both plexus, and tractographies were performed. The disease histories and the Inflammatory Rasch-built Overall Disability Scale (I-RODS) questionnaire scores were documented before MRI scans.

**Results:**

The sizes of nerve roots were larger in CIDP (*p* < 0.01). CR, nT2, and ADC were significantly higher, while FA was lower in CIDP than in controls (*p* < 0.01). FA had the highest sensitivity (0.809) and area under the curve (AUC) (0.925), while the highest specificity was 0.961 for CR in single parameters. The combination of FA and CR has the highest sensitivity, specificity, accuracy, and AUC in the LS plexus. CR only had a weak correlation with nT2 (*p* < 0.05). ADC and diameter had a positive correlation with nT2, and the diameter and nT2 had a negative correlation with FA in CIDP (*p* < 0.05). FA had a negative correlation with the duration in the CIDP (*r*’s = −0.404, *p* < 0.05). There was no significant correlation between the I-RODS scores and MR multiparameters (*p* < 0.05).

**Conclusion:**

Multisequence MRN possesses a high diagnostic performance in the LS plexus. Sampling perfection with application-optimized contrasts using different flip angle evolutions (SPACE) combined with DTI and contrast enhancement serves as a recommended composite protocol for CIDP.

## Introduction

Chronic inflammatory demyelinating polyneuropathy (CIDP) is an acquired immune-mediated and treatable demyelinating disorder of the peripheral nervous system (Rotta et al., 2000; Vallat et al., 2010; Latov, 2014). CIDP is most frequently observed in adult men and has an annual incidence of 0.48 per 100,000 individuals in the population (Iijima et al., 2008). The diagnosis and management of CIDP can be difficult and are mainly based on clinical features and nerve conduction studies (Latov, 2014; Querol et al., 2017; Rajabally et al., 2017). However, CIDP is characterized clinically by heterogeneous, sensory, and motor impairment with a chronic progressive or relapsing–remitting course (Rotta et al., 2000; Alabdali et al., 2017). Therefore, patients frequently undergo electrophysiology, lumbar puncture examinations, and at times even nerve biopsy for establishing the diagnosis. However, a major limitation of neuroelectrophysiology is the inability to assess nerves at all sites, especially the proximal nerve roots of the deep plexus that are often involved (Lichtenstein et al., 2018).

Hypertrophy with T2 signal alterations or gadolinium enhancement of peripheral nerves has been described on MRI in patients with CIDP, but it is only a supportive criterion (level C recommendation) according to the 2010 guidelines of the European Federation of Neurological Societies/Peripheral Nerve Society (Van den Bergh et al., 2010). Conventional MR neurography provides only a view of the limited region of nerve trunks, or a description of the morphological and signal abnormalities. More commonly, MR neurography, based on heavily T2-weighted imaging with fat suppression combined with T1-weighted sequences, is used to assess peripheral neuropathy (Takahara et al., 2008; Yoshida et al., 2015). However, this may not enough identify the condition of complicated diseases, especially when hypertrophy has not yet appeared in the peripheral nerves at the early stage. CIDP is considered to be an autoimmune disease involving the cellular and humoral immune responses. Gadolinium enhancement on MRI may also work by indicating the presence of an inflammatory process, as occurs in CIDP (Duggins et al., 1999). However, quantitative studies on the enhancement of the nerves are rarely done.

In this study, we have implemented a quantitative multisequence protocol for brachial and lumbosacral (LS) plexus neurography to gain a multifaced biological characterization of peripheral nerve tissues. Concurrently, the potential correlation between parameters and their diagnostic efficiency were investigated to come up with optimal combined sequences.

## Materials and Methods

### Subjects

The study was performed with the approved by the ethics review committee (No. IORG0003571) and was registered on ClinicalTrials.gov (ChiCTR1800016450).

All subjects signed a written informed consent prior to enrolment. The study was conducted from March 2016 to September 2019. Thirty-seven newly diagnosed patients (newly diagnosed and re-diagnosed cases, who were previously misdiagnosed) with typical CIDP were recruited from the Neuromuscular Center of our hospital. The inclusion criteria for CIDP are follows: typical CIDP; a neurologist verified that their conditions met the European Federation of Neurological Societies/Peripheral Nerve Society diagnostic criteria; spectrum of typical CIDP; and definite chronic progressive and relapsing courses only. The disease histories and the Inflammatory Rasch-built Overall Disability Scale (I-RODS) questionnaire scores, which have proved as an outcome measurement for assessing activity limitations, were documented before MRI scans individually in patients. Additionally, 37 healthy controls who matched patients with CIDP in age (±2 years) and gender were recruited from our institution. Exclusion criteria included any contraindication to MRI and the presence of renal insufficiency.

### Magnetic Resonance Neurography

MR neurography was performed on a 3-T whole-body MR system (MAGNETOM Trio, Siemens Healthcare, Erlangen, Germany). A four-channel neck and contiguous three-multichannel body matrix coils linked to the spine element coils were employed from the skull base to the upper thigh.

The MRI protocol of the brachial and LS plexus included volumetric interpolated breath-hold examination (VIBE), turbo inversion recovery magnitude (TIRM), and three-dimensional (3D) sampling perfection with application-optimized contrasts using different flip angle evolutions (SPACE) sequences on the coronal plane and the single-shot echo-planar-imaging (EPI)-based DTI sequence on the axial plane. The multi-sequences of the brachial and LS plexus were scanned separately. First, TIRM, VIBE, and DTI sequences were performed. Then, a macrocyclic gadolinium-based contrast agent (Gadovist, Bayer Healthcare, Leverkusen, Germany) was injected intravenously at a dose of 0.1 ml/kg with a flow rate of 1.5 ml/s. Finally, contrast-enhanced (ce)-VIBE was scanned immediately after the injection of a contrast agent, then 3D SPACE was applied. The center k-space line of ce-VIBE was located at 80 s. Specific sequence parameters are shown in Supplementary Table 1. The duration of each scan session was 50 min.

### Image Post-processing and Analysis

Post-processing of raw MRI data and DTI analysis were performed with software provided by the MR system manufacturer (Syngo MR Workspace, Siemens Healthcare). Qualitative and quantitative assessments were performed independently by two experienced radiologists (J.W., > 10 years of neuroimaging experience; X.S., > 3 years of neuroimaging experience), who were blinded to the clinical information. The final averaged values were obtained by three repeated measurements in each parameter. Furthermore, one of the radiologists (X.S.) carried out a second-time quantitative assessment after 12 weeks. Two radiologists qualitatively assessed for abnormal findings (hypertrophy or/and hyperintensity/contrast enhancement) of the brachial and LS plexus with blind, separately. Abnormal MR findings of diffusion tensor tractography were recorded. For any disagreements between the two radiologists in the qualitative assessments, the final conclusion was taken by consensus.

### Quantitative Analysis

#### Nerve Diameters

The diameters of the nerve roots at C5-C8 and L4-S1 levels were measured perpendicular to their long axes, at 1.0 cm away from the dorsal root ganglion (DRG) on both sides on the maximum intensity projection (MIP, 15 slices and thickness was 15.0 mm) images from the 3D SPACE image. The diameters of the bilateral sciatic and femoral nerves were determined at the upper edges of the femoral heads in the coronal and sagittal planes, respectively.

#### Contrast-Enhanced Ratios

The contrast-enhanced ratios (CR) of bilateral C5-C8 and L4-S1 nerve roots were determined at the same location by copying the ROI between the ce VIBE and non-ce VIBE sequences, at 1.0 cm away from the DRG. The background noise was measured outside of the body region. The ce nerve signal-to-noise ratio (ce nSNR) = nerve signal intensity _ce VIBE_/background noise _ce VIBE_. The non-ce nSNR = nerve signal intensity _non–ce VIBE_/background noise _non–ce VIBE_. The CR was defined as CR = ce nSNR/non-ce nSNR.

#### Nerve-to-Muscle T2 Signal Intensity Ratios

The nerve-to-muscle T2 signal intensity ratios (nT2) were obtained by placing the ROI on bilateral C5-C8 and L4-S1 nerve roots and calculated in relation to the adjacent deep cervical and iliopsoas muscles on the TIRM sequence. The nT2 was defined as the following: nT2 = nerve root T2 signal intensity/adjacent muscle T2 signal intensity.

#### DTI Parameters

The average apparent diffusion coefficient (ADC) and fractional anisotropy (FA) values were calculated on the DTI by hand-operated circular ROI in an anatomical b0 field image that was drawn beyond 1.0 cm of the DRG at the bilateral C5-C8 and L4-S1 nerve roots. For diffusion tensor tractography, a turning angle of 30° and an FA threshold of 0.10 were employed. The step size of the deterministic tractography algorithm was 1.0 mm.

Nerve diameters were reported separately for the C5-C8 and L4-S1 nerve roots. Also, the CR, nT2 values, and DTI parameters were represented by a value for the brachial plexus and LS plexus, respectively, as a whole.

### Quality Analysis

The image quality was evaluated based on the degree and uniformity of fat suppression and the presence of motion artifacts affecting the nerve visualization. It was scored on a scale from 1 to 3 (1: excellent, 2: moderate, 3: poor) on morphological sequences, including VIBE, TIRM, and SPACE. The DTI quality was assessed depending on the presence of motion artifacts and distortion, (1) excellent, artifact-free or a little; (2) moderate, some artifacts or distortion not affecting measurement; and (3) poor, severe artifacts and image distortions. Images with “poor” classification were excluded.

### Statistical Analysis

Statistical analysis was performed by IBM SPSS statistical software version 25 (IBM Corp., Armonk, NY, United States) and GraphPad Prism 7.0 (GraphPad software, San Diego, CA, United States). Categorical variables were summarized as frequencies and proportions. The median (M) and interquartile range (IQR) were expressed in non-normal distributed continuous data. The Wilcoxon signed-rank test was used to assess the differences in each parameter between the patients and controls. ROC curve analysis was used to determine the diagnostic efficiency. The joint analysis of the MR parameters was conducted using a binary logistical regression, and the generated variable probability was fitted to the joint curve of ROC. Correlation or partial correlation analyses after adjustment for gender and age were performed using Spearman’s correlation coefficients. Intraclass correlation coefficients (ICC) were calculated to the consistency of the inter- and intra-observer. Multiple-hypothesis testing was addressed with Bonferroni correction. Two-tailed *p* < 0.05 was considered statistically significant.

## Results

A total of 74 subjects (37 patients with CIDP, 37 age- and gender-matched controls) were studied. The clinical characteristics are provided in Table 1.

**TABLE 1:** Clinical characteristics^1^.

	CIDP	Control	*p value*
Total number	37	37	n/a
Age (years)	51 (19–71)	50 (20–69)	0.832
Weight (kg)	62.4 (16.5)	65.3 (12.3)	0.478
Height (cm)	168.1 (16.9)	165.4 (15.4)	0.380
Gender (male/female)	26/11	26/11	n/a
The period^a^ (Months)	18 (7, 36)	n/a	n/a
Dominant involved extremities (L/U/L&U)	16/7/14	n/a	n/a
Treatment (S/IVIG)	8/5	n/a	n/a
Treatment duration	7 (8), *n* = 10	n/a	n/a
I-RODS score	35.5 (13.5)	n/a	n/a

*^1^CIDP, chronic inflammatory demyelinating polyneuropathy; I-RODs, Rasch-built Overall Disability Scale; U, upper limbs; L, lower limbs; S, steroids; n/a, not available. The numbers in parentheses indicate the interquartile range. ^a^The period between the onset of initial symptoms and the confirmed diagnosis (M).*

### Quality Assessments

The quality assessment of the brachial and LS plexus achieved the rating of “excellent” in over half of the images (Supplementary Table 2). One of the patients with CIDP only performed the brachial plexus imaging and did not complete the LS plexus imaging due to the involuntary movements of the feet. Therefore, the LS plexus images of this patient and the paired control were excluded. One brachial VIBE image and DTI images of brachial and LS plexus were rated as “poor” qualities and were excluded with the corresponding control images (Supplementary Table 2).

### Images and Qualitative Analysis

Abnormal findings (nerve hypertrophy or/and hyperintensity/contrast enhancement) were noticed in the brachial plexus of 22/37 (59.5%) and in the LS plexus of 27/36 (75.0%) of the patients with CIDP, and in none of the healthy controls (Supplementary Figure 1). The coherence rate of the two radiologists was 96% (72/75) in the qualitative assessments.

**FIGURE 1:**
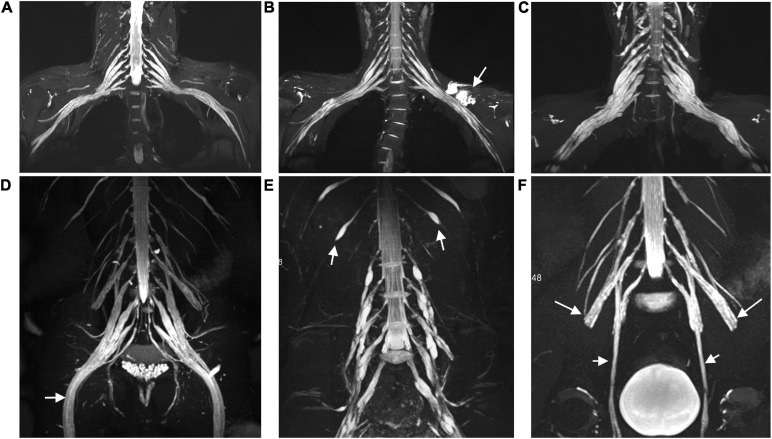
Representative coronal MIP images (SPACE) show diffuse uniform and multifocal fusiform hypertrophy of the brachial **(A–C)** and lumbosacral **(D–F)** plexus in patients with CIDP. The sciatic (arrow in **D**), intercostal (arrow in **E**), femoral (long arrows in **F**), and obturator nerve (arrows in **F**) involvements present diffuse symmetrical hypertrophy. A patient with a 7-year disease course showing uniform thickening in the brachial plexus **(B)**, but multifocal fusiform hypertrophy in the lumbosacral plexus **(E)**. The lesion with a long T2 signal on the left side represents a subcutaneous hemangioma (arrow in **B**). Image **(C)** shows multifocal fusiform hypertrophy with striking increased T2 signal intensity in the brachial plexus.

**FIGURE 2:**
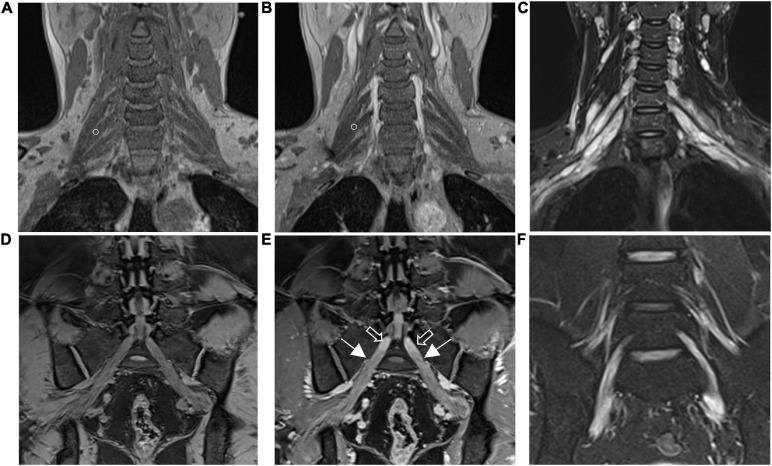
The brachial **(A,B)** and lumbosacral **(D,E)** plexus on non-contrast-enhanced volumetric interpolated breath-hold examination (VIBE) **(A,D)** and contrast-enhanced VIBE sequences **(B,E)**, respectively. Images show the right C7 nerve root with absence of enhancement (circles in **A,B**). Bilateral S1 nerve roots demonstrate obviously enhanced (arrows in **E**) and vividly enhancing ganglia (hollow arrows in **E**). Images on turbo inversion recovery magnitude (TIRM) show a striking increased T2 signal intensity in the brachial **(C)** and lumbosacral **(F)** plexus. The CR, ce nSNR/non-ce nSNR; SNR, nerve signal intensity/background noise; ce, contrast-enhanced; nT2, nerve root T2 signal intensity/adjacent muscle T2 signal intensity.

**FIGURE 3:**
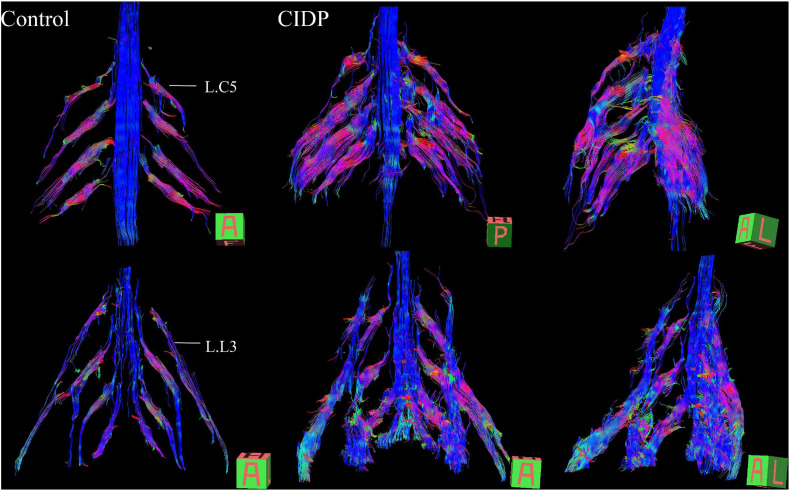
Diffusion tensor tractography (DTT) of healthy control and CIDP patients shows the bundles of plexus nerve fiber and their branches in three-dimensional views. Tractography shows thickened, partially discontinuous, and distorted fiber tracts of the brachial and lumbosacral plexus and their branches in patients with CIDP. A, anterior view; P, posterior view; A–L, left-frontal oblique view. The main dominant direction of diffusion is color-coded (blue: *z*-axis = nerve course; red: *x*-axis (left/right); green: *y*-axis (up/down).

Hypertrophy and enhancement were illustrated as seen in the brachial and LS plexus MR neurography of the patients with CIDP (Figures 1, 2). The thickening, irregularity, and partial discontinuity of the brachial and LS plexus were found on diffusion tensor tractography in patients with CIDP (Figure 3). The reconstructed fiber bundles of the plexus in controls are also shown in Figure 3. Representative *B*-value = 0 image, ADC, and FA maps of nerve roots in patients with CIDP are exhibited in Supplementary Figure 2.

### Quantitative Analysis

**FIGURE 4:**
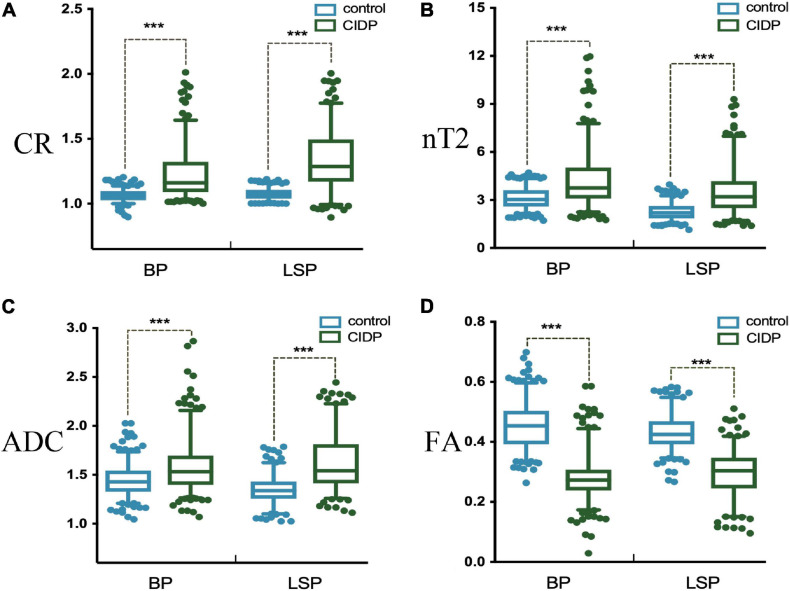
Contrast-enhanced ratio **(A)**, nerve-to-muscle T2 signal intensity ratio **(B)**, and apparent diffusion coefficient **(C)** of the brachial and lumbosacral nerve roots were significantly higher in patients with CIDP than in controls. Fractional anisotropy **(D)** of the brachial and lumbosacral nerve roots was significantly lower in patients with CIDP than in controls. *p < 0.05; ***p < 0.001; BP, brachial plexus; LSP, lumbosacral plexus.

The diameters of the C5-C8 and L4-S1 nerve roots and sciatic and femoral nerves were significantly larger in patients with CIDP than in healthy controls (all *p* < 0.001, Supplementary Table 3 and Supplementary Figure 3). The CR, nT2, and ADC of the brachial and LS nerve roots were significantly higher in patients with CIDP than in controls, while FA was lower in CIDP (*p* < 0.01, Figure 4 and Table 2).

**TABLE 2:** Multiparameters for brachial and LS plexi^2^.

		CR	nT2	FA	ADC
BP	CIDP	1.16 (0.23)	3.75 (1.94)	0.273 (0.09)	1.533 (0.302)
	Control	1.06 (0.07)	3.06 (1.03)	0.454 (0.112)	1.339 (0.289)
LSP	CIDP	1.29 (0.32)	3.21 (1.68)	0.303 (0.103)	1.541 (0.400)
	Control	1.07 (0.07)	2.20 (0.78)	0.425 (0.044)	1.339 (0.177)

*^2^BP, brachial plexus; LSP, lumbosacral plexus; CIDP, chronic inflammatory demyelinating polyneuropathy; CR, contrast-enhanced ratio; nT2, nerve-to muscle T2 signal intensity ratio; FA, fractional anisotropy. The numbers in parentheses indicate the interquartile range.*

### Analysis of Diagnostic Accuracy

**FIGURE 5:**
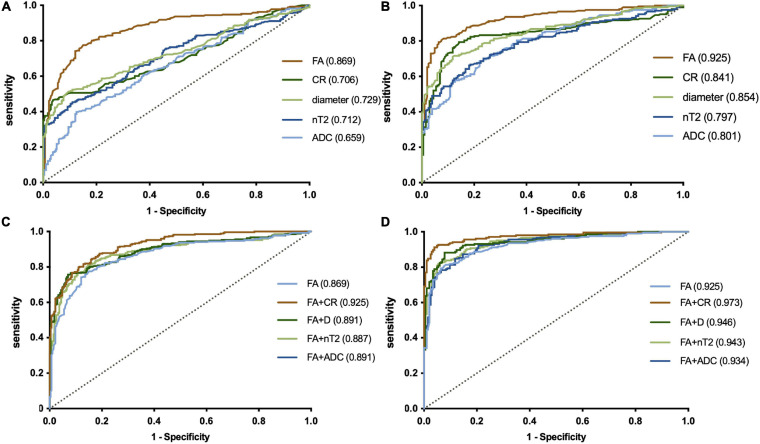
ROC plots revealing the performance of a single-parameter model in the plexus (**A**, brachial plexus; **B**, LS plexus) and the combined model in the plexus (**C**, brachial plexus; **D**, LS plexus). The largest AUC was 0.925, using the cutoff value of 0.361 for the fractional anisotropy (FA) of the LS plexus in a single-parameter model **(B)**. The combined analysis demonstrated that the combination of FA and the contrast-enhanced ratio has the largest AUC (0.973) in the LS plexus **(B)**. LS, lumbosacral. The numbers in parentheses indicate the respective AUC values.

In a single-parameter model, FA had the largest area under the curve (AUC) (0.925) using a cutoff value of 0.361, with the highest sensitivity (80.9%) and accuracy (0.73) in the LS plexus (Table 3 and Figure 5), while the highest specificity was 96.1% given by CR in the LS plexus (Table 3). The AUC, sensitivity, specificity, and accuracy of the combined two-parameter analysis are provided in Supplementary Table 3. The combined analysis of FA and CR demonstrated the highest AUC (0.973), sensitivity (92.2%), specificity (96.3%), and accuracy (0.885) in the LS plexus, which have significant differences among each pair, respectively (Figure 5 and Supplementary Tables 4, 5).

**TABLE 3:** ROC curve analysis on single-parameter model^3^.

		D	nT2	CR	ADC	FA
AUC	BP	0.729	0.712	0.706	0.659	0.872
	LSP	0.854	0.797	0.841	0.801	0.925
Sensitivity (%)	BP	51.3	45.4	46.5	39.9	77.5
	LSP	71.6	66.7	72.5	68.6	80.9
Specificity (%)	BP	90.5	89.0	93.3	87.5	85.0
	LSP	87.7	81.4	96.1	77.9	92.2
Youden’s index	BP	0.418	0.344	0.428	0.274	0.625
	LSP	0.593	0.481	0.686	0.465	0.731

*^3^D, diameter; nT2, nerve-to muscle T2 signal intensity ratio; CR, contrast-enhanced ratio; FA, fractional anisotropy; BP, brachial plexus; LSP, lumbosacral plexus.*

### Correlations With MR Parameters

Both the diameter and nT2 had moderate negative correlations with FA in patients with CIDP (*| r*’s*|* = 0.41–0.52; *p* < 0.05) and corresponding weak or no correlations in controls (Table 4). Both the ADC and diameter had moderate positive correlations with nT2 in patients with CIDP (*| r*’s*|* = 0.51–0.61; *p* < 0.05) and corresponding weak positive or no correlations in controls (Table 4). There was a moderate negative correlation between FA and ADC (*| r*’s*|* = 0.40–0.50; *p* < 0.01) and a weak positive correlation between diameter and ADC both in patients with CIDP and controls (*| r*’s*|* = 0.35–0.38; *p* < 0.01). CR had a weak correlation with nT2 and no significant correlation with other parameters in patients with CIDP and controls (Table 4).

**TABLE 4:** Spearman correlation coefficients among multiparameters^4^.

		**FA&D**	**FA&nT2**	**FA&CR**	**FA&ADC**	**CR&nT2**

*r*’s (BP)	CIDP	−0.435^∗∗^	−0.412^∗∗^	0.183	−0.454^∗∗^	0.216^∗∗^
	Control	−0.166^∗∗^	−0.119^∗^	−0.066	−0.403^∗∗^	0.111^∗^
*r*’s (LSP)	CIDP	−0.520^∗∗^	−0.455^∗∗∗^	0.085	−0.498^∗∗∗^	0.156^∗^
	Control	−0.002	−0.051	0.021	−0.412^∗∗^	0.162^∗^

		**nT2&ADC**	**D&nT2**	**CR&ADC**	**CR&D**	**D&ADC**

*r*’s (BP)	CIDP	0.511^∗∗^	0.578^∗∗^	−0.135	−0.176	0.377^∗∗^
	Control	0.215^∗∗^	0.119^∗^	−0.089	−0.201	0.348^∗∗^
*r*’s (LSP)	CIDP	0.605^∗∗^	0.509^∗∗^	−0.109	0.077	0.362^∗∗∗^
	Control	0.099	0.040	0.010	−0.064	0.358^∗∗^

^4^r’s, Spearman correlation coefficient; BP, brachial plexus; LSP, lumbosacral plexus; FA, fractional anisotropy; CR, contrast-enhanced ratio; nT2, nerve-to muscle T2 signal intensity ratio; D, diameter; CIDP, chronic inflammatory demyelinating polyneuropathy; *p < 0.05; **p < 0.01; ***p < 0.001.

### Correlations Between Clinical and MR Parameters

There was a moderate negative correlation between FA value with the course duration in the CIDP patients (*r*’s = −0.404, *p* < 0.05; Table 5). There was, however, no significant correlation between the I-RODS scores and MR multiparameters.

**TABLE 5:** Correlations between clinical and MR parameters^5^.

	Duration		I-RODs	
	*r*’s	*P*^#^	*r*’s	*p*^#^
D	0.214	0.233	0.019	0.912
nT2	0.049	0.785	0.169	0.347
CR	−0.020	0.910	−0.151	0.404
FA	−0.406	0.016^∗^	−0.066	0.716
ADC	0.124	0.480	−0.173	0.339

^5#^, Age- and sex-adjusted. r’s, Spearman correlation coefficient; p, p value; I-RODS, inflammatory Rasch-built overall disability scale; D, diameter; nT2, nerve-to muscle T2 signal intensity ratio; CR, contrast-enhanced ratio; FA, fractional anisotropy. MR parameters defined as the mean of brachial (bilateral C5–C8), lumbosacral (bilateral L4–S1) nerve roots. *p < 0.05.

### Inter- and Intra-Observer Performance

There were good inter- and intra-observer consistencies for each parameter assessed in the individual nerve roots. The ranges of the intraclass correlation coefficients (inter- and intra-observer) were 0.823–0.934 for the diameter in C5-C8, L4-S1, sciatic, and femoral nerve roots and 0.781–0.873 for CR, 0.792–0.853 for nT2, 0.732–0.859 for FA, and 0.729–0.823 for ADC in C5-C8 and L4-S1 nerve roots, respectively.

## Discussion

We here present a novel multiparametric MR imaging paradigm that allows the simultaneous quantifications of the architectural configuration and microstructural properties in the brachial and LS plexus. Our data not only confirmed the findings that the diameter, nT2, and ADC of nerve roots are increased while FA is reduced in CIDP patients but also helped to analyze the diagnostic performance separately and in combination. FA of the LS plexus possessed the highest sensitivity and accuracy, while CR had the highest specificity in the single-parameter model. Furthermore, the combination of FA and CR had the best diagnostic efficiency. Nonetheless, our results do not considerably differ to those by Breckwoldt et al. (2015) who suggested that FA combined with nT2 had the highest diagnostic efficiency in peripheral polyneuropathy. This discrepancy may be due to the fact that contrast enhancement has not been incorporated, and the enrolment of patients with various etiologies probably was not the representative of CIDP entity.

Several previous studies have described the diffuse swelling and high signal intensity on T2-weighted images in nerve trunks and plexus (Goedee et al., 2017; Hiwatashi et al., 2017; Jomier et al., 2020). We observed that brachial, LS plexus, and their branches showed uniform symmetrical or multifocal fusiform thickening in patients with CIDP. Our findings confirmed that quantitative analysis performs better compared to qualitative description. Hypertrophy of the nerves can be attributed to the inflammatory infiltrate and onion-bulb formation due to repeated demyelination and remyelination (Ginsberg et al., 2004). Our study provides proof for a moderate correlation of nerve diameters and nT2/DTI parameters in our cohort, which support this hypothesis. Once an onion-bulb hypertrophy has been formed, it is rarely resorbed by treatment.

The reported increased T2 signal in CIDP is plausible given the pathologic processes with the interstitial edema, increased myelin membranes, or macromolecules by remyelination (Piccione et al., 2016; Rajabally et al., 2017). It is suggested that nT2 may be helpful for the inflammatory neuropathy prognosis evaluation in longitudinal studies, as it is sensitive to the intra-neural water content (Kronlage et al., 2017a). By the use of a CIDP model, non-obese diabetic mice, it was demonstrated that nT2 could be used as a marker for monitoring the response to treatment (Meyer Zu Horste et al., 2016), and that more clinical studies are needed.

It is generally acknowledged that the DTI is a non-invasive functional MR for peripheral nerve, which depicts the microstructural integrity of neural tissue (Kronlage et al., 2017b). Previous studies have exclusively reported DTI parameter alterations in the limb nerves in patients with CIDP (Kakuda et al., 2011; Lichtenstein et al., 2018); our study assesses the diagnostic accuracy of DTI with tractography in the brachial and LS plexus, which complements with the literature. A previous study found that abnormalities are present in both the proximal and distal nerves, and the demyelination is more pronounced proximally than distally (Markvardsen et al., 2016; Lehmann et al., 2019). Some researchers also suggested that the exploration of proximal nerve segments can be particularly useful in diseases with multifocal demyelinating lesions such as CIDP (Hughes et al., 2001; Vallat et al., 2010). Therefore, DTI has become important in the assessment of proximal nerve segments that cannot be evaluated by standard electrophysiological methods or ultrasound (Kakuda et al., 2011). Of note is that the FA threshold of the CIDP patients should to be lower than that of healthy subjects in tractography. When below the threshold (we set the FA value to 0.1), fiber tracking will be interrupted.

Brachial EPI-based DTI is susceptible to respiratory motion artifacts. The optimal cutoff value in our cohort was 0.36 for an average FA of the nerve roots, which is lower than the one given by Markvardsen et al. (2016) who defined a cutoff value of 0.45 of the sciatic nerve to identify CIDP. This could be that the measurements are depending on not only the nerves but also the segments and the sequence parameters. Previous studies have reported that FA gradually decreased with increasing age (Guggenberger et al., 2012). To avoid this potential age-related confounder, we included age-matched controls.

The blood–nerve barrier (BNB) defines the intra-neural microenvironment of the peripheral nervous system. These tight junctions, especially the innermost casing connections, provide a barrier to the diffusion of various tracers with larger molecular weight, such as contrast agents (Olsson and Reese, 1971). In the peripheral nerves of CIDP patients, increased permeability of the BNB appears to be the cause of spinal roots and cauda equina enhancement. Early studies have indicated a decreased number of tight-junction proteins claudin 5 and ZO-1 in the sural nerve biopsy specimens of CIDP patients (Kanda et al., 2004; Vallat et al., 2010). These findings may suggest that BNB not only has increased permeability but also has been damaged. Gadolinium enhancement has been detected in some, but not all, cases in inflammatory demyelinating neuropathies (Duggins et al., 1999; Tsuchiya et al., 2007). We reported the utility of contrast enhancement to quantify the alteration of the BNB permeability in the plexus. Our results suggest that the CR has the advantage of high specificity, but with relatively poor sensitivity. At present, there is no consensus that the enhancement indicates disease activity in researches (Kuwabara et al., 1997; Albulaihe et al., 2016; Decard et al., 2018). CR is likely to be a potential non-invasive repeatable MR biomarker for detecting and monitoring disease activity, which needs to be further worked on.

In our result, the diameter and nT2 had a moderate negative correlation with FA, and the diameter and ADC had a moderate positive correlation with nT2 in CIDP but had a corresponding weak or no correlation in controls. These results can be attributed to the pathophysiological basis of inflammatory cell infiltration, interstitial edema, repeated demyelination, and remyelination, which are reflected by multiple parameters. The moderate positive correlation of ADC with nT2 could be due to the relatively high *b*-value chosen in this study (T2 shine-through effect due to low SNR in b 900). CR only had a weak correlation with nT2 and no correlation with other parameters, likely suggesting a representation of specific pathophysiological changes that would serve as a complementary MR biomarker to DTI.

Duration of disease showed a significantly negative correlation with mean FA. This indicates that the FA reflects the severity of nerve injury with disease duration to some extent. A few studies have suggested a negative association between FA and clinical outcome, including Inflammatory Neuropathy Cause and Treatment (INCAT) and Neuropathy Impairment Score (NIS) (Mathys et al., 2013; Markvardsen et al., 2016). However, we found no clear correlation between I-RODS with any MR parameters, which may require more trials with larger samples.

### Limitations

Our study has several limitations. First, we did not directly correlate CR with disease activity, because it is difficult to obtain histologic results in all patients in our hospital. Second, some of the patients in our cohort have received therapy (steroids or IVIG) which may underestimate the diagnostic efficiency of MR examination; however, our result was still satisfactory. Moreover, it is hard to know whether prior drug treatment yet has any influence on the current state of this rare disease, which is mostly characterized by chronic progressive and relapsing courses. Third, quantitative or semiquantitative parameters, such as CR and nT2, would be influenced to some extent by sequence/scanner type/field strength, etc., so this needs to be interpreted with caution. The fourth is the lack of disease controls and patients with mimics of CIDP such as other chronic demyelination peripheral neuropathies, which limits the diagnostic specificity. The need persists for a differential diagnosis that distinguishes patients with CIDP or some of the diseases also harbor a similar clinical presentation and/or morphologic abnormalities.

## Conclusion

In conclusion, we present a novel multiparametric MRI protocol that allows non-invasive quantification of the brachial and LS plexus nerve abnormality in CIDP patients. 3D SPACE with high-resolution MR neurography, combined with DTI and contrast enhancement, is a recommended composite protocol in diagnosis and management of CIDP.

## Data Availability Statement

The raw data supporting the conclusions of this article will be made available by the authors, without undue reservation.

## Ethics Statement

The studies involving human participants were reviewed and approved by the present prospective study was approved by the Ethics Committee of Tongji Medical College, Huazhong University of Science and Technology (No. IORG0003571). The patients/participants provided their written informed consent to participate in this study.

## Author Contributions

CZ, XS, and XK conceived and designed. XS and JW analyzed the data. XS, CZ, ZL, and OA manuscript preparation. HZ and XK contributed reagents, materials, and analysis tools. All authors contributed to the article and approved the submitted version.

## Conflict of Interest

HZ was employed by the company Siemens Healthineers, Shanghai, China. The remaining authors declare that the research was conducted in the absence of any commercial or financial relationships that could be construed as a potential conflict of interest.

## Publisher’s Note

All claims expressed in this article are solely those of the authors and do not necessarily represent those of their affiliated organizations, or those of the publisher, the editors and the reviewers. Any product that may be evaluated in this article, or claim that may be made by its manufacturer, is not guaranteed or endorsed by the publisher.
