# Supplementary material for: Multisequence Quantitative Magnetic Resonance Neurography of Brachial and Lumbosacral Plexus in Chronic Inflammatory Demyelinating Polyneuropathy
Source: Front Neurosci. 2021 Jul 23;15:649071. doi: 10.3389/fnins.2021.649071 (PMC8346234; doi:10.3389/fnins.2021.649071)
Supplement: Supplementary file 1 [file Data_Sheet_1.DOCX]

**Supplementary Table 1. Magnetic resonance sequence parameters^1^**

| Sequences | TR  (ms) | TE  (ms) | Avg | Slices | AT  (min) | FOV  (mm^2^) | Voxel  (mm^3^) | BW  (hz/Px) | Other |
| --- | --- | --- | --- | --- | --- | --- | --- | --- | --- |
| SPACE | 3000 | 270 | 1.8 | 144 | 10′50 | 448×448 | 1.0×1.0×1.0 | 425 | IPAT-3 |
| TIRM | 4000 | 39 | 2 | 37 | 4′20 | 448×448 | 1.5×1.2×4.0 | 250 | IPAT-2 |
| VIBE | 8.18 | 3.04 | 1.8 | 120 | 2′40 | 448×448 | 1.0×1.0×1.0 | 130 | IPAT-2 |
| DTI | 6000 | 92 | 4 | 45 | 5′32 | 256×256 | 2.0×2.0×3.0 | 1346 | B-0;900s/mm^2^  DOI-20 IPAT-2 |

^1^ TR: repetition time; TE: echo time; Avg: average; AT: acquisition time; FOV: field of view; BW: bandwidth; Px: pixel; B: diffusion moment; DOI: directions of interrogation; IPAT: integrated parallel acquisition technique; SPACE: sampling perfection with application-optimized contrasts using different flip angle evolution; TIRM: turbo inversion recovery magnitude; VIBE: volumetric interpolated breath-hold examination; DTI: diffusion tensor imaging.

| Sequence | CIDP | | | |  |  | Control | | | |
| --- | --- | --- | --- | --- | --- | --- | --- | --- | --- | --- |
|  |  | total | excellent | moderate | poor |  | total | excellent | moderate | poor |
| SPACE | BP | 37 | 27 | 10 | 0 |  | 37 | 25 | 12 | 0 |
|  | LSP | 36 | 24 | 12 | 0 |  | 36 | 26 | 10 | 0 |
| VIBE | BP | 37 | 23 | 13 | 1 |  | 37 | 24 | 13 | 0 |
|  | LSP | 36 | 26 | 10 | 0 |  | 36 | 27 | 9 | 0 |
| TIRM | BP | 37 | 28 | 9 | 0 |  | 37 | 27 | 10 | 0 |
|  | LSP | 36 | 28 | 8 | 0 |  | 36 | 28 | 8 | 0 |
| DTI | BP | 37 | 19 | 17 | 1 |  | 37 | 21 | 16 | 0 |
|  | LSP | 36 | 23 | 12 | 1 |  | 36 | 23 | 13 | 0 |

**Supplementary Table 2. The quality assessment of plexi^2^**

^2^CIDP: chronic inflammatory demyelinating polyneuropathy; BP: brachial plexus; LSP: lumbosacral plexus; SPACE: sampling perfection with application-optimized contrasts using different flip angle evolution; VIBE: volumetric interpolated breath-hold examination; TIRM: turbo inversion recovery magnitude; DTI: diffusion tensor imaging.

**Supplementary Table 3. Brachial and lumbosacral plexi nerve roots diameter (mm)^3^**

|  | CIDP | Control | *P* value |  | CIDP | Control | *P* value |
| --- | --- | --- | --- | --- | --- | --- | --- |
| C5 | 4.43 (1.42) | 3.68 (0.71) | < 0.001 | L4 | 6.24 (1.78) | 4.94 (0.73) | < 0.001 |
| C6 | 5.11 (1.89) | 4.70 (0.78) | < 0.001 | L5 | 8.38 (3.35) | 6.02 (1.07) | < 0.001 |
| C7 | 5.40 (1.82) | 4.67 (0.88) | < 0.001 | S1 | 7.52 (3.87) | 5.29 (0.44) | < 0.001 |
| C8 | 5.18 (1.57) | 4.21 (0.64) | < 0.001 | SN | 14.47 (4.11) | 9.73 (1.18) | < 0.001 |
| na | na | na | na | FN | 6.43 (2.64) | 4.58 (1.16) | < 0.001 |

^3^ CIDP: chronic inflammatory demyelinating polyneuropathy; SN: sciatic nerve; FN: femoral nerve; The numbers in parentheses indicate the interquartile range.

|  |  | FA+D | FA+nT2 | FA+CR | FA+ADC | CR+nT2 | CR+D | nT2+D |
| --- | --- | --- | --- | --- | --- | --- | --- | --- |
| AUC | BP | 0.891 | 0.887 | 0.925 | 0.891 | 0.885 | 0.855 | 0.766 |
|  | LSP | 0.946 | 0.943 | 0.973 | 0.934 | 0.966 | 0.964 | 0.879 |
| Sensitivity  (%) | BP | 75.7 | 80.9 | 79.8 | 75.7 | 63.2 | 70.6 | 55.9 |
|  | LSP | 88.2 | 82.4 | 92.2 | 84.8 | 91.7 | 88.7 | 72.5 |
| Specificity  (%) | BP | 93.0 | 85.3 | 89.0 | 93.0 | 94.9 | 90.1 | 93.4 |
|  | LSP | 92.2 | 94.1 | 96.3 | 88.2 | 92.6 | 96.1 | 94.6 |
| Youden’s index | BP | 0.687 | 0.662 | 0.688 | 0.687 | 58.1 | 60.7 | 49.3 |
|  | LSP | 0.804 | 0.765 | 0.885 | 0.730 | 0.843 | 0.848 | 0.671 |

**Supplementary Table 4.** **ROC curve analysis on two-parameter model^4^**

^4^ AUC: area under the ROC curve; FA: fractional anisotropy; D: diameter; nT2: nerve-to muscle T2 signal intensity ratio; CR: contrast-enhanced ratio; ADC: apparent diffusion coeffecient; BP: brachial plexus; LSP: lumbosacral plexus;

**Supplementary Table 5. The difference of the ROC curve analysis on two-parameter model in Lumbosacral plexus ^5^**

| **Joint analysis** |  |  | **95% Confidence Interval** | |
| --- | --- | --- | --- | --- |
|  | ***p*** | **χ** | Lower | Upper |
| **FA+CR- FA+D** | 0.0223^*^ | 5.2227 | .0039 | .0507 |
| **FA+CR- FA+nT2** | 0.0049^**^ | 7.9015 | .0093 | .0519 |
| **FA+CR -FA+ADC** | 0.0002^***^ | 13.6591 | .0187 | .0610 |
| **FA+D – FA+ nT2** | 0.6693 | 0.1825 | -.0118 | .0184 |
| **FA+D – FA+ADC** | 0.1225 | 2.3847 | .0034 | .0285 |
| **FA+nT2– FA+ADC** | 0.922 | 2.88351 | -.0015 | .0201 |
| **FA-FA+CR** | <.0001^****^ | 17.5112 | -.0717 | -.0260 |
| **FA- FA+D** | 0.0045^**^ | 8.0682 | -.0364 | -.0061 |
| **FA- FA+nT2** | 0.0033^**^ | 8.6460 | -.0304 | -.0061 |
| **FA-FA+ADC** | 0.0643 | 3.4231 | -.0185 | .0005 |

^5^FA: fractional anisotropy; CR: contrast-enhanced ratio; nT2: nerve-to muscle T2 signal intensity ratio; D: diameter; CIDP: chronic inflammatory demyelinating polyneuropathy; *: *P* < 0.05; **: *P* < 0.01; ***: *P* < 0.001.

**Supplementary Figure 1**


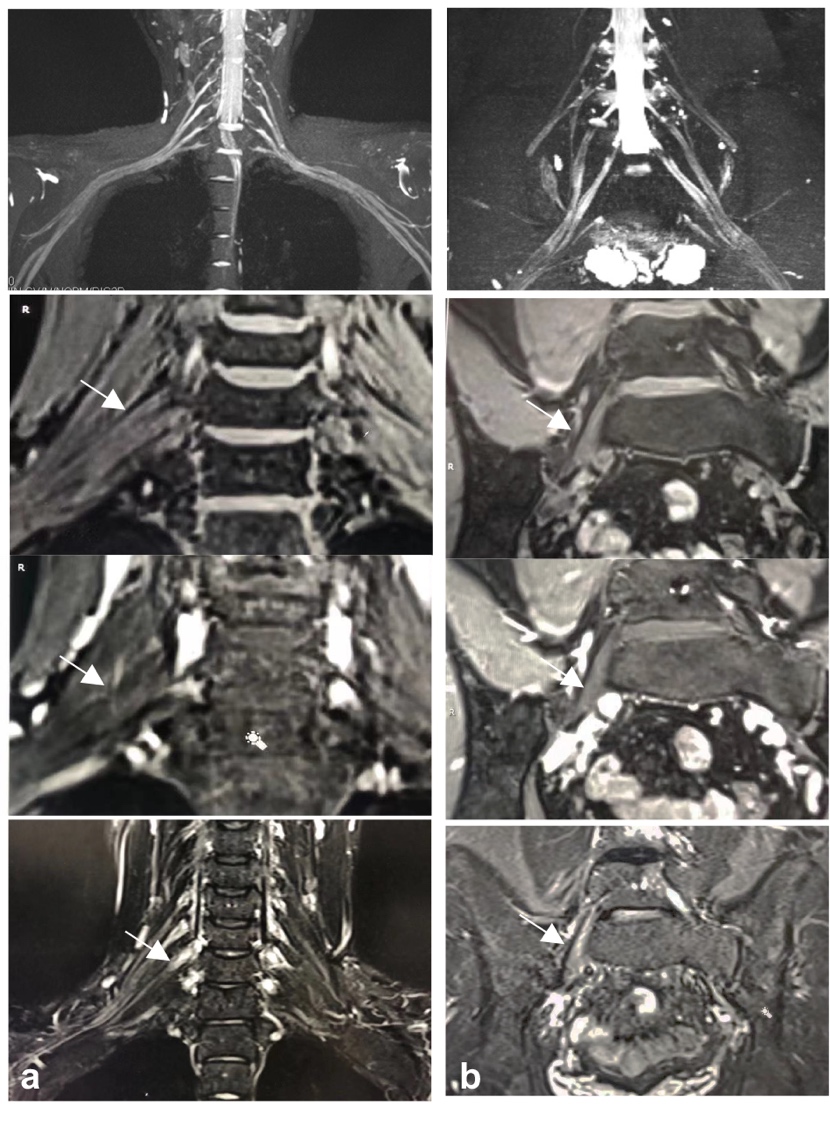


Figure S.1 Normal control. Brachial (a) and lumbosacral (b) plexus show the symmetrical uniform signal intensities without enhancement of nerve roots. The sequences on the left corner of images. The arrow refers to the represented right C7 nerve root.


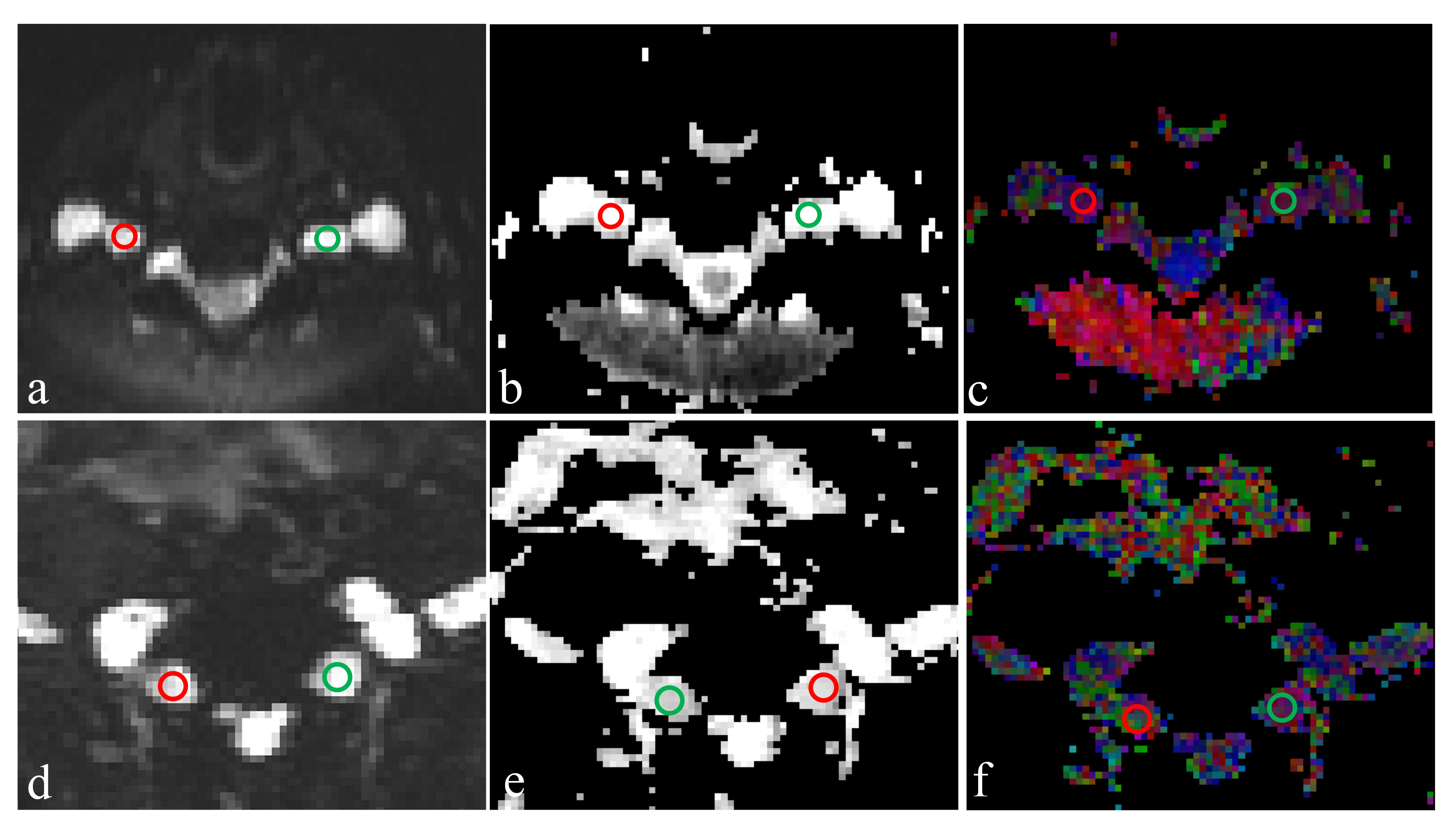
**Supplementary Figure 2**

Figure S.2 Representative cross-sectional B0, grayscale ADC and color FA maps of the brachial (a-c; C5 nerve root level; circles) and lumbosacral plexus (d-f; S1 nerve root level; circles) in patients with CIDP. Note the nerve root swelling and signal intensity increase on B0 map (a, d). ADC shows increased diffusion (b, e) and FA exhibit decreased anisotropy (c, f) in the involved nerves.

**Supplementary Figure 3**


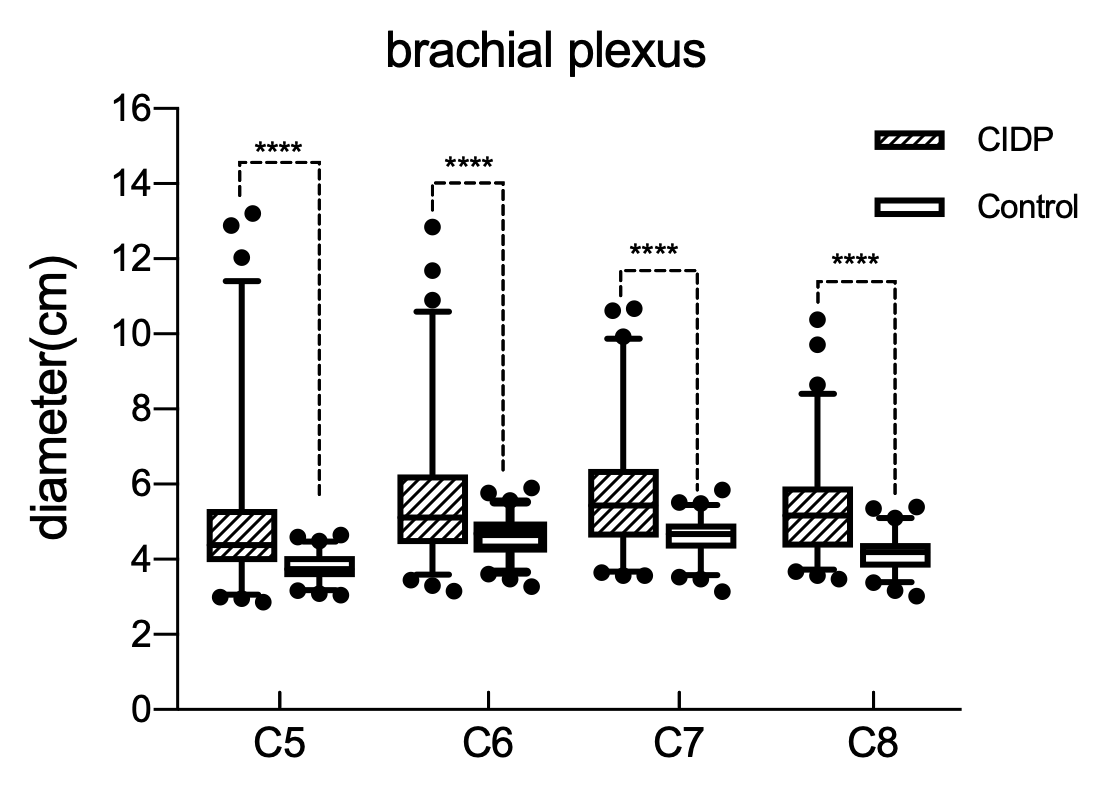

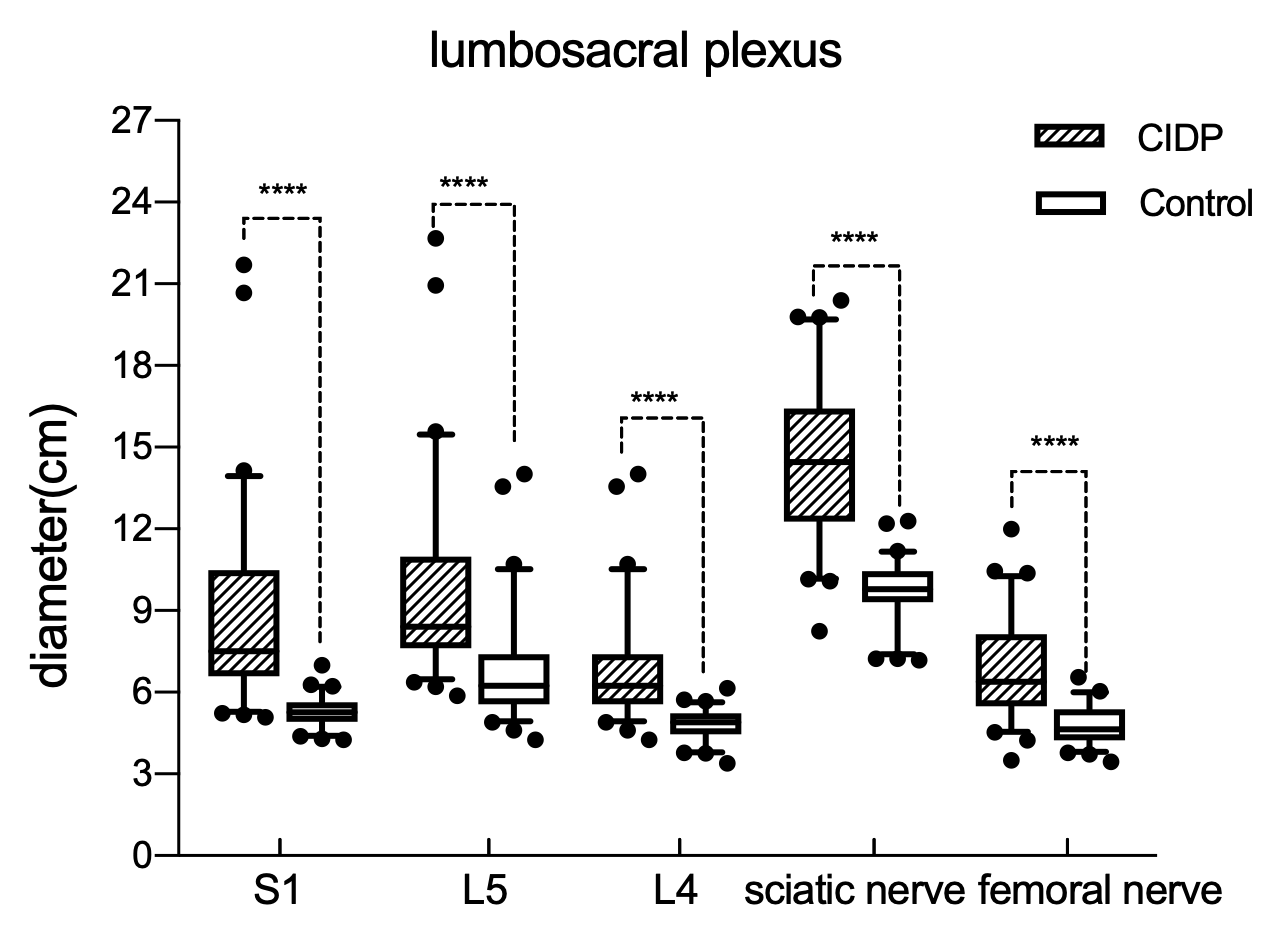


Figure S.3 Diameter’s boxplots. The diameters of brachial and lumbosacral nerve roots were significantly higher in patients with CIDP than in controls. *: P < 0.05; ***: P < 0.001; BP: brachial plexus; CIDP: chronic inflammatory demyelinating polyneuropathy.
